# The effects of exploratory behavior on physical activity in a common animal model of human disease, zebrafish (*Danio rerio*)

**DOI:** 10.3389/fnbeh.2022.1020837

**Published:** 2022-11-08

**Authors:** Cairsty DePasquale, Kristina Franklin, Zhaohan Jia, Kavya Jhaveri, Frances E. Buderman

**Affiliations:** ^1^Department of Biology, Pennsylvania State University – Altoona, Altoona, PA, United States; ^2^Department of Ecosystem Science and Management, Pennsylvania State University, University Park, PA, United States

**Keywords:** open field, novel tank diving test, exploratory behavior, physical activity, swimming, zebrafish

## Abstract

Zebrafish (*Danio rerio*) are widely accepted as a multidisciplinary vertebrate model for neurobehavioral and clinical studies, and more recently have become established as a model for exercise physiology and behavior. Individual differences in activity level (e.g., exploration) have been characterized in zebrafish, however, how different levels of exploration correspond to differences in motivation to engage in swimming behavior has not yet been explored. We screened individual zebrafish in two tests of exploration: the open field and novel tank diving tests. The fish were then exposed to a tank in which they could choose to enter a compartment with a flow of water (as a means of testing voluntary motivation to exercise). After a 2-day habituation period, behavioral observations were conducted. We used correlative analyses to investigate the robustness of the different exploration tests. Due to the complexity of dependent behavioral variables, we used machine learning to determine the personality variables that were best at predicting swimming behavior. Our results show that contrary to our predictions, the correlation between novel tank diving test variables and open field test variables was relatively weak. Novel tank diving variables were more correlated with themselves than open field variables were to each other. Males exhibited stronger relationships between behavioral variables than did females. In terms of swimming behavior, fish that spent more time in the swimming zone spent more time actively swimming, however, swimming behavior was inconsistent across the time of the study. All relationships between swimming variables and exploration tests were relatively weak, though novel tank diving test variables had stronger correlations. Machine learning showed that three novel tank diving variables (entries top/bottom, movement rate, average top entry duration) and one open field variable (proportion of time spent frozen) were the best predictors of swimming behavior, demonstrating that the novel tank diving test is a powerful tool to investigate exploration. Increased knowledge about how individual differences in exploration may play a role in swimming behavior in zebrafish is fundamental to their utility as a model of exercise physiology and behavior.

## Introduction

Animal models, in particular rodents, are often used to fill in the gaps in our knowledge where studies on humans are lacking. Much research has been conducted on individual differences in exploration and activity level in rodents [see review by Gosling (2001)]. However, there is limited research on how individual differences in exploration affect physical activity in non-human animals. Most rodent studies of exercise and behavior utilize voluntary wheel-running, which is rewarding for rodents and represents self-motivating behavior (Novak et al., 2012). Where the interaction of individual differences in exploration with exercise has been explored, results have been contradictory; high levels of exploration in the open field test have been associated with increased wheel running in mice (Careau et al., 2012), however, mice bred for increased wheel running displayed similar levels of activity in the open field compared with control mice (Bronikowski et al., 2001; Careau et al., 2012).

Zebrafish (*Danio rerio*) are a well-known animal model of human disease, but only recently has their potential as a model for exercise physiology and behavior come to light (Luchiari and Chacon, 2013; Gilbert et al., 2014; DePasquale and Leri, 2018). Zebrafish represent a superior alternative for studying the interaction between exploration and physical activity because they are cheap and easy to maintain, they possess many homologous genes to humans, and many of their neuroanatomical pathways resemble that of the mammalian brain. Moreover, a number of studies have already documented the occurrence of individual differences in exploration in zebrafish, and there are a number of standard assays for measuring it (Blaser and Gerlai, 2006; Blaser and Rosemberg, 2012; Maximino et al., 2012; Stewart et al., 2012). However, despite these advantages and the increased use of zebrafish in biomedical research, the effects of individual differences in exploration on physical activity have not yet been explored in zebrafish.

Traditionally, individual variation exhibited by animal models was avoided in the biomedical sciences because behavioral deviation from the norm was viewed as noise (Monaghan, 2014). More recently, researchers have acknowledged the importance of individual variation as a factor underlying an animal’s capacity to respond to environmental demands, however, there is still considerable debate about how to properly define and measure these behavioral traits (Koski, 2011). Therefore, it has been suggested that a triangulation of approaches (using multiple behavioral tests of the same construct) is a useful tool for increasing the robustness of findings (Carter et al., 2013; Fontana et al., 2022).

Thus, the current study aimed to enhance our understanding of the connection between exploration and physical activity in zebrafish. In an attempt to use a triangulation of approaches, we included two different tests to measure exploration in zebrafish; the open field test and the novel tank diving test. The open field test was originally developed in rodents and has been adapted for use on fish to measure levels of boldness, thigmotaxis, and exploration (Dahlbom et al., 2011; Stewart et al., 2012). The novel tank diving test is a standard test which exploits the stress response in fish to determine anxiety-like behaviors such as exploration, diving, and freezing (Blaser and Rosemberg, 2012). Therefore, both the open field and novel tank diving test have been used independently as measures of exploration in zebrafish, but the validation of using one of these behavioral tests over the other is unknown. In order to quantify physical activity, we looked at voluntary access to a flow of water as a means of motivation to exercise. Specifically, we were interested in understanding if a fish that chose to be near a flow of water would also engaged in more swimming behavior. Due to high levels of dependency among variables, we used regression trees to determine the variables that were best at predicting activity levels. We hypothesize that behavior in the open field and novel tank tests would be strongly correlated. We also predicted that bolder, more exploratory, less anxious fish would spend more time in an area of flowing water.

## Materials and methods

### Animals

All experimental and husbandry procedures were approved by the Pennsylvania State University’s Animal Care Committee (protocol 201900937). Nine-month old wild-type (AB) zebrafish (*n* = 48) were purchased from Penn State College of Medicine, Hershey and were randomly distributed across eight home tanks (40 cm × 20 cm × 26 cm) with equal sex ratio (3:3). Each home tank had a biofilter, heater, gravel substrate, two plastic plants and a small plastic shelter. The fish were maintained on a 12 L: 12 D cycle with simulated dawn and dusk periods, and a water temperature of 25 ± 1°C. The fish were fed once daily with commercial flake food (TetraMin^®^ Tropical Flakes) and live cultures of brine shrimp and were maintained in these conditions for 3 months prior to the start of behavioral trials. Behavioral trials were conducted over 8 weeks; fish from one home tank (*n* = 6) were screened in both exploration assays and observed in the choice tank each week. All behavioral assays were conducted in daylight conditions.

### Open field test

On day one each week, all fish from one home tank were screened for exploratory/boldness behaviors in the open field test. The open-field test arena consisted of a clear, plastic tank (28 cm × 32 cm × 15 cm) with water depth 8 cm covered on all sides with black plastic. Three replicate arenas were constructed to allow testing of multiple fish at the same time. The arenas were placed on the floor and were positioned in such a way that no direct light fell on the arenas and no shadows were created by the arena walls. At the start of each trial, a fish was carefully netted from their home tank and placed in a transparent plastic cylinder (diameter 10 cm) in the center of the arena. After 2 min, the cylinder was removed, and behavior was recorded for 5 min using a video camera on a tripod positioned to film the open field arena from above. At the end of the trial, the fish was gently netted out of the open field arena and placed in a holding tank separate from the home tank. The water in the open field arena was replaced with sump water of the same temperature as the home tank before the next fish was tested.

### Novel tank diving test

On the same day following the open field tests, all fish from the same home tank were then screened for anxiety-like behaviors in the novel tank diving test. The novel tank diving arena consisted of a plain tank (35 cm × 19 cm × 28 cm) surrounded by black plastic on three sides and filled with water to a depth of 24 cm. Again, three replicate novel tank diving arenas were used to allow testing of multiple fish at the same time. The tanks were placed on a table and a camera on a tripod facing the uncovered side of the tank recorded behavior. An individual fish was carefully netted from their home tank and gently placed in the water at the top of the novel tank diving test arena. When first released, all fish swam to the bottom of the tank, which is stereotypical behavior in the novel tank diving test (Cachat et al., 2010). Data collection began once the fish reached the bottom of the tank, and the fish was free to explore the novel tank for 5 min. At the end of the observation period, the fish was netted from the novel tank and placed in an experimental tank. The water in the novel tank was replaced with sump water of the same temperature as the home tank before the next fish was tested.

### Experimental procedure for exercise observations

**FIGURE 1:**
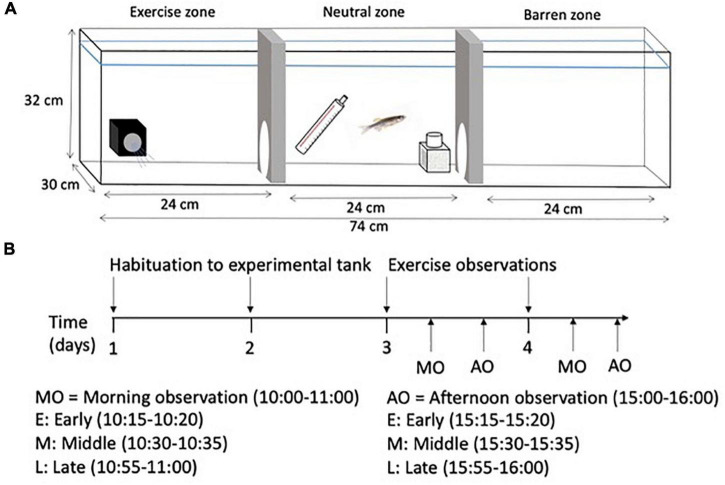
**(A)** Schematic diagram of the experimental tank showing the exercise zone with the submersible pump, the neutral middle zone with the heater and biofiler, and the barren zone, and **(B)** a timeline showing the habituation period and the observation periods in the experimental tank. Data collected during each 5-min segment included: Data collected during all 5-min segments: Total time in swim zone, no. of entries into swim zone, total time spent swimming, total time in barren zone, and no. of entries into barren zone. For recursive partitioning, the average value across all timepoints was calculated for each variable (one value per individual and variable).

Once all fish from a home tank had been screened for exploration, they were placed in individual experimental tanks (Figure 1A). Six experimental tanks (74 cm × 30 cm × 32 cm; water depth 26 cm) were covered with black plastic on three sides and were divided into three equal compartments (length: 24 cm): an exercise zone containing a submersible pump (Song Long Submersible Pump, SL-381, Chicago, IL, USA; water flow set to 0.1 m/s), a barren zone (no flow), and a neutral middle zone where food was delivered. The neutral zone contained a heater and a biofilter. The walls between the zones contained a small opening (9 cm × 9 cm) through which the fish could freely move between zones. Individual fish were placed in the tank to acclimate to their surrounding for 2 days and were fed flake food in the neutral zone during regular feeding hours. After a 2-day acclimatization period, a camera on a tripod was used to record the behavior of each fish for 2 h each day for 2 days: 1 h at 10:00 and 1 h at 15:00 (Figure 1B). Once all recordings had been completed, each fish was netted from its experimental tank and placed back in its home tank. The experimental tank water was replaced with sump water in preparation for the next fish.

### Video analysis

All videos were analyzed using Behavioral Observation Research Interactive Software, BORIS (Friard and Gamba, 2016) and acetate placed over the video image on the computer screen. To evaluate zebrafish swimming activity and exploratory behavior in the open field test, the acetate was composed of a 4 cm × 4 cm grid which included a line drawn 4 cm from the perimeter from the tank to measure thigmotaxic behavior. Several variables were calculated from the raw data including latency to the edge zone, proportion of time spent in the center zone, mean duration of visits to the center zone, proportion of time frozen (where freezing behavior was defined as the fish was immobile for >1 s), and the rate of movement (number of lines crossed/s). The values for each individual fish across each variable can be found in Supplementary Figure 1. To evaluate vertical and horizontal swimming activity in the novel tank diving test, the acetate was composed of a 3 cm × 3 cm grid and three vertical zones (bottom, middle, and top). Several variables were again calculated from the raw data including latency to top, total time spent in the top, number of entries into the top, number of entries top/bottom (indicative of the relative switch rate between zones), average time spent in the top per entry, duration of time spent frozen (where freezing behavior was defined as the fish was immobile for >1 s), and movement rate (number of lines crossed/s). The values for each individual fish across each variable can be found in Supplementary Figure 2. To make manual behavioral scoring more manageable, three 5-min timeslots were analyzed (15–20, 30–35, and 55–60 min) of each 1-h observation in the experimental tanks (Figure 1B) to determine how much time the fish spent in the exercise zone versus the barren zone and how many times the fish entered into each zone. The amount of time the fish spent actively swimming (rostral end pointed toward the flow of water coming from the pump) was also recorded. The average value (across individuals) for each exercise variable at each time-point and a comparison of the average exercise variables (across time) for time in swim zone and time spent swimming for each individual can be found in Supplementary Figures 3, 4.

### Statistical analysis

#### Exploration tests

We calculated the Pearson correlation coefficient for individual pair-wise combinations of variables across the open field and novel tank diving tests to determine relationships between behavioral measures of exploration. We used the size and direction of the correlation coefficient as the foundation of our inference about potential relationships; however, we also used an α-value of 0.05 to ensure we observed statistical significance between independent pairs of variables that we discuss. We expected some measures within the open field and novel tank diving tests to be positively or negatively related because of their implicit relationship to one another (Supplementary Table 1). Correlations were visualized using the corrplot package (Wei and Simko, 2021).

#### Exercise observations

We calculated the Pearson correlation coefficient for individual pair-wise combinations of exercise variables using the average across sessions (i.e., each individual was represented by the means of their exercise variables across sessions). We also calculated the correlation coefficients between each of two exercise variables of particular interest, time in swim zone and time spent swimming, and the open field and novel tank diving variables. In addition, we evaluated the consistency of individual behavior by examining the Pearson correlation coefficients for each variable through time. We used the magnitude and direction of the correlation coefficient as the foundation of our inference about relationships between variables; however, we present the statistical significance of the correlations using an α-value of 0.05 between independent pairs of variables in order to supplement the information on magnitude and directionality. Three individuals with missing data during some sessions due to recording issues were removed from the analysis. Correlations were visualized using the corrplot package (Wei and Simko, 2021).

We then used recursive partitioning, specifically regression trees, to predict the average time an individual spent in the swim zone and actively swimming using the exploration variables as predictors (Breiman et al., 1984). Recursive portioning is a type of machine learning algorithm that is used for classification and prediction. Machine learning methods are useful for situations in which predictors are correlated with other predictors, the sample size is small relative to the number of potential predictors, and when there might be complex non-linearities in the relationships between predictors and response variables [as reviewed in Strobl et al. (2009)]. These methods offer an alternative to principal component analyses (PCA), in which the predictor variables are projected into a reduced set of variables; although PCA allows for the use of correlated predictor variables and is a method of dimension reduction, the method comes at a cost because the individual effects of predictor variables are no longer identifiable [as reviewed in Strobl et al. (2009)]. Regression trees determine an optimal set of binary splits that can be used to predict the response variable; each split is based on a predictor variable and a threshold value of the predictor variable. The split can be chosen a number of ones, but one of the common methods for continuous response variables is to select the split that maximizes the between-groups sum-of-squares.

All individuals (*n* = 48), were used for the machine learning analysis because we used the average values of the exercise variables across time. We used regression trees due to the highly correlated set of exploration variables and because our goal was to classify which behavioral variables were best able to predict exercise levels, not necessarily assuming a mechanistic relationship between them (Buston and Elith, 2011). We fit the regression trees in R (R Core Team, 2022) using the rpart package (Therneau and Atkinson, 2022) and visualized the results using the rpart.plot package (Milborrow, 2021). We did not allow any terminal nodes to have fewer than six fish in them (the number of fish per tank). We relied on the terminal node size restriction to limit the size of our tree, as opposed to selecting an optimal complexity parameter based on *k*-fold cross-validation.

## Results

### Tests of exploration

#### Correlations within the novel tank diving test

**FIGURE 2:**
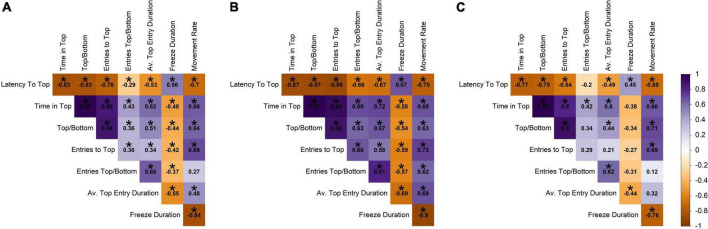
Pearson correlation among novel tank variables across **(A)** all individuals, **(B)** males, and **(C)** females. Darker shades of purple indicate strong positive correlations, darker shades of orange indicate strong negative correlations, and white indicates no correlation. Statistically significant correlations (α = 0.05) are indicated with *.

The novel tank diving variables were correlated with themselves at an absolute value of 0.27–0.97 (raw range −0.84–0.97; Figure 2A). For example, fish that took longer to reach the top in the novel tank diving test spent less time in the top, had fewer entries into the top, had a shorter average top entry duration, had a slower movement rate, and spent more time freezing. Time in the top of the tank was most strongly correlated with other novel tank diving variables, with an average absolute correlation of 0.69.

Interestingly, the novel tank diving variables were more strongly correlated overall in males than in females (Figures 2B,C). For example, latency to top was more negatively correlated with time in top/bottom and entries top/bottom for males (−0.87 and −0.66) than females (−0.75 and −0.2). In addition, there was a weak relationship between movement rate and entries top/bottom in females (0.12), but these variables had a strong positive relationship in males (0.62). Relationships that were statistically significant at an α-value of 0.05 are denoted with an asterisk and are presented as information to supplement the interpretation of the direction and magnitude of the correlation; however, there were small correlation coefficients that we did not discuss that were determined to be significant.

#### Correlations within the open field test

**FIGURE 3:**
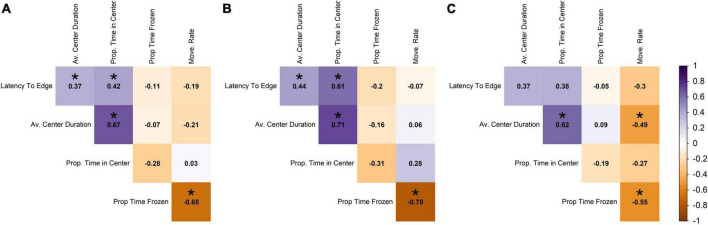
Pearson correlation among open field variables across **(A)** all individuals, **(B)** males, and **(C)** females. Darker shades of purple indicate strong positive correlations, darker shades of orange indicate strong negative correlations, and white indicates no correlation. Statistically significant correlations (α = 0.05) are indicated with *.

In contrast to the novel tank diving variables, the open field variables had low correlation among themselves, with absolute values 0.03–0.68 (raw range −0.68–0.67; Figure 3A). The highest correlated open field variables were average center duration and proportion of time in center (0.67; the more time in the center, the longer the average center duration), and proportion of time frozen and movement rate (−0.68; the more time frozen, the lower the movement rate; Figure 3A).

In terms of the open field data for males, the proportion of time spent frozen in the open field was highly negatively correlated with movement rate (−0.78; Figure 3B), and proportion of time in center was highly positively correlated with average duration in the center of the tank (0.71; Figure 3B), but these correlations were weaker in the females (−0.55 and 0.62, respectively; Figure 3C). Males and females differed in the direction of correlation between movement rate and average center duration and proportion time in center, with males showing positive correlations and females showing negative (with movement rate and average center duration being significantly negative for females; Figures 3B,C). Relationships that were statistically significant at an α-value of 0.05 are denoted with an asterisk and are presented as information to supplement the interpretation of the direction and magnitude of the correlation; however, there were small correlation coefficients that we did not discuss that were determined to be significant.

#### Correlations between the novel tank diving test and open field test

**FIGURE 4:**
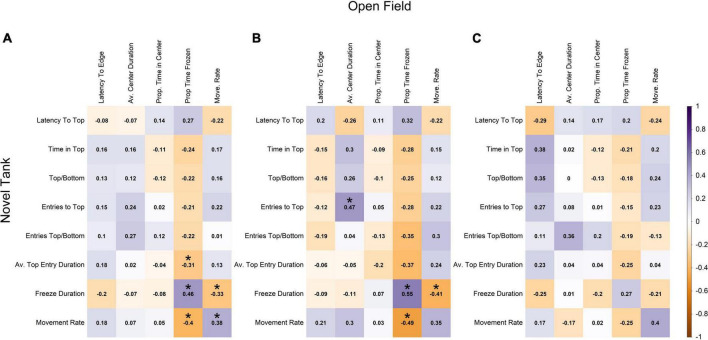
Pearson correlation among each novel tank and open field variable across **(A)** all individuals, **(B)** males, and **(C)** females. Darker shades of purple indicate strong positive correlations, darker shades of orange indicate strong negative correlations, and white indicates no correlation. Statistically significant correlations (α = 0.05) are indicated with *.

There were also low levels of correlation between the sets of novel tank and open field variables. The two largest positive correlations between the two sets of variables were between freezing duration in the novel tank diving test and the proportion of time frozen in the open field test (0.46), and movement rates between the two tests (0.38; Figure 4A). The two largest negative correlations involved the same variables; freezing duration in the novel tank diving test was negatively correlated with movement rate in the open field test (−0.33), and movement rate in the novel tank diving test was negatively correlated with proportion of time frozen in the open field test (−0.40; Figure 4A).

When the data was split into separate sex classes (Figures 4B,C), the data again followed a similar pattern with very little correlation between novel tank diving variables and open field variables. For both sexes, the novel tank diving variables were more correlated with each other than the open field variables were with each other. However, males showed a slightly stronger positive relationship between freezing duration in the novel tank and proportion of time frozen in the open field (0.55 vs. 0.27; Figures 4B,C) and a slightly more negative correlation between movement rate in the novel tank and proportion of time frozen in the open field than females (−0.49 vs. −0.25; Figures 4B,C). There were also sign differences between the two sexes, in which two variables were positively correlated for one sex but negatively correlated with the other, but they were limited to comparisons among open field variables and between novel tank and open field variables (i.e., no sign difference among novel tank diving variables; Figures 4B,C). Relationships that were statistically significant at an α-value of 0.05 are denoted with an asterisk and are presented to support the relationships discussed previously; however, there are small correlation coefficients that we did not discuss that were determined to be significant.

### Exercise observations

#### Correlations between exercise variables

**FIGURE 5:**
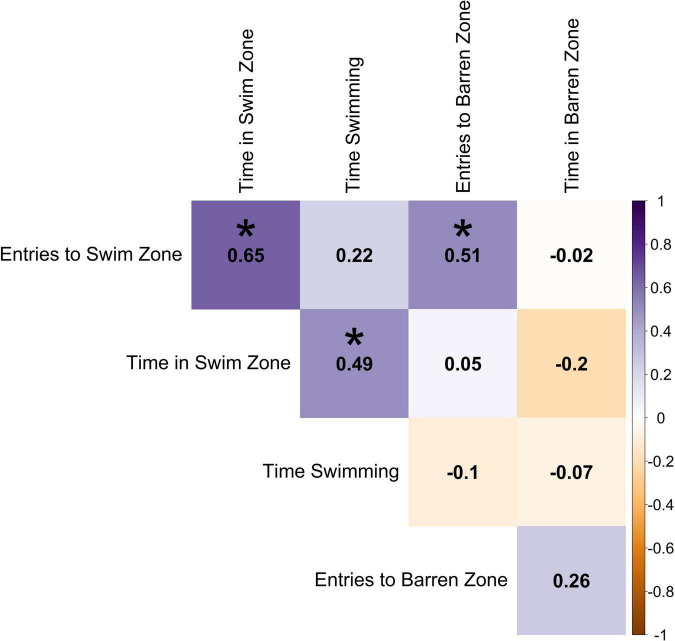
Pearson correlation between the individual averages of exercise variables across sessions. Darker shades of purple indicate strong positive correlations, darker shades of orange indicate strong negative correlations, and white indicates no correlation. Statistically significant correlations (α = 0.05) are indicated with *.

Using the average exercise variables for each individual, entries into swim zone had a positive correlation with time in swim zone (0.65) and entries into barren zone (0.51), however, there was no correlation with time in barren zone (−0.02; Figure 5). Time in barren zone had a weak negative correlation with time in swim zone (−0.2; the more time a fish spent in the swim zone, the less time it spent in the barren zone). Time spent swimming was positively correlated with time spent in swim zone, although this was a relatively weak association (0.49).

**FIGURE 6:**
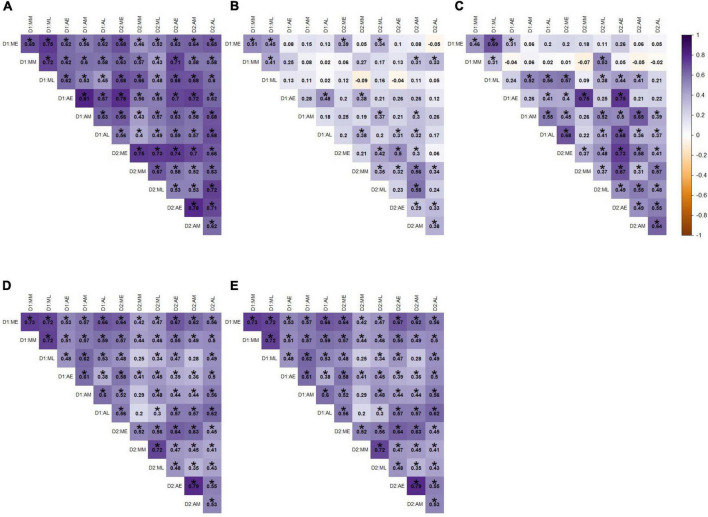
Pearson correlation coefficients for exercise variables across all time points for **(A)** entries into swim zone **(B)** time spent in swim zone, **(C)** time spent swimming, **(D)** entries into barren zone, and **(E)** time spent in barren zone. Labels correspond to the day of the session (D1 or D2), morning or afternoon session (M or A), and within morning or afternoon, whether it was the early, middle, or late session (E, M, or L). Darker shades of purple indicate strong positive correlations, darker shades of orange indicate strong negative correlations, and white indicates no correlation. Statistically significant correlations (α = 0.05) are indicated with *.

In terms of the exercise variables across all time points for all individuals with complete sets of observations, the number of entries into swim zone was highly correlated across the course of the experiment (Figure 6A). Thus, on average fish were consistently crossing into the swim zone at a similar rate across all time points. Similarly, number of entries into the barren zone was weakly correlated across the course of the experiment (Figure 6D). Interestingly, there was very little correlation across time points for time spent in swimming zone and time spent in barren zone (Figures 6B,E). Thus, fish were inconsistent in the amount of time they spent in either zone, with time spent in swimming zone even being negatively correlated with itself. Time spent swimming was more consistent across time points on day 2 of testing (Figure 6C; as depicted by the darker tones of purple in the lower half of the Figure).

#### Correlations between exploration and exercise variables

The Pearson correlation coefficient suggests that the novel tank diving variables correlate better with time in swim zone (mean absolute value 0.35) and time spent swimming (mean absolute value 0.25) than open field variables (0.13 and 0.09, respectively; Table 1). However, overall, all variables showed weak correlations (all coefficients <0.5; Table 1). Fish that took less time to reach the top, spent more time in the top, had more entries into the top, spent longer on average in the top, froze less and spent more time moving in the novel tank diving test spent more time in the swim zone and more time swimming (though this was a weaker relationship).

**TABLE 1:** Pearson correlation coefficients of exercise variables against novel tank and open field personality variables.

Personality variables	Time swim zone	Time swimming
NT: Latency to top	–0.47	–0.19
NT: Time in top	0.43	0.32
NT: Top bottom	0.43	0.28
NT: Entries to top	0.34	0.25
NT: Entries top bottom	0.38	0.26
NT: Av. top entry duration	0.36	0.37
NT: Freeze duration	–0.21	–0.18
NT: Move. rate	0.22	0.17
OF: Latency to edge	0.18	0.02
OF: Av. center duration	0.27	0.13
OF: Prop. time center	0.05	0.10
OF: Prop. time frozen	0.01	–0.11
OF: Move rate	–0.17	0.04

#### Recursive partitioning

**FIGURE 7:**
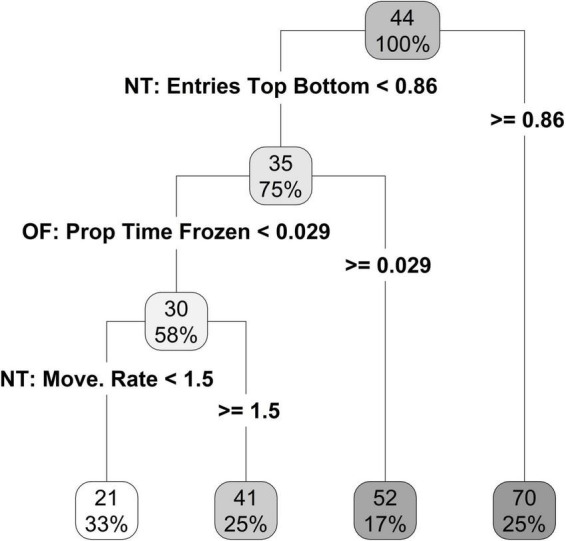
Regression tree, limited to a terminal node size of six individuals, for time spent in the swim zone using the novel tank and open field personality variables.

Two novel tank variables, entries top/bottom and movement rate, and one open field variable, proportion of time frozen, were selected by the recursive partitioning process as good predictors of the average time an individual spent in the swim zone (Figure 7). The first best predictor for average time in swim zone was the proportion of entries into the top/bottom in the novel tank; individuals that made more than 0.86 entries top/bottom had a predicted value of 70 s on average in the swim zone (25% of individuals) and those that made less than 0.86 entries into top/bottom spent 35 s on average in the swim zone (75% of individuals; Figure 7). Given an individual made fewer entries into the top/bottom and they spent more than 0.029 s frozen, then the predicted average time in the swim zone was 52 s (17% of individuals; Figure 7). If individuals made fewer entries to the top/bottom and spent a short amount of time frozen (the majority of individuals), then slower moving individuals (those with a movement rate less than 1.5) tended to spend less time in the swim zone (Figure 7).

**FIGURE 8:**
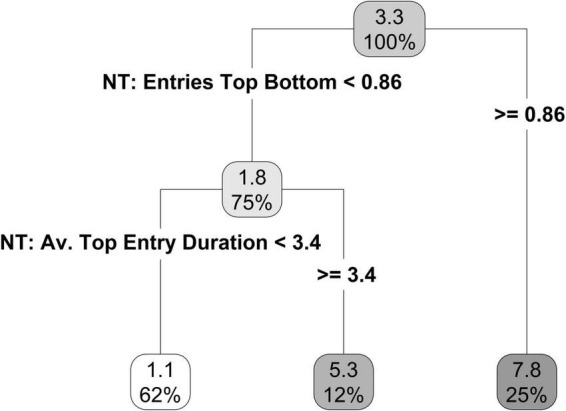
Regression tree, limited to a terminal node size of six individuals, for time spent swimming using the novel tank and open field personality variables.

Recursive partitioning identified two best predictors of time spent swimming (Figure 8). Similar to time spent in the swim zone, the first best predictor was entries top/bottom in the novel tank diving test. If an individual made more than 0.86 entries top/bottom, then they had a predicted average swim time of 7.8 s (25% of individuals; Figure 8). If an individual had fewer than 0.86 entries top/bottom, then they had a predicted average swim time of 1.8 s (75% of individuals; Figure 8). The second-best predictor was the average top entry duration, in which individuals who spent shorter periods of time entering the top of the tank also spent less time swimming (Figure 8).

## Discussion

Contrary to our predictions, the correlation between novel tank diving test variables and open field test variables was relatively weak (no correlation was less than −0.5 or more than 0.5). Novel tank diving variables were more correlated with themselves than open field variables were to each other. The data followed a similar pattern when it was split into the different sex classes, but males exhibited stronger relationships between variables than did females. In terms of exercise variables, fish that spent more time in the swimming zone spent more time actively swimming, however, exercise behavior was inconsistent across the time of the study. Although our results suggest the novel tank diving variables more accurately predicted time in swimming zone and time spent actively swimming, all relationships with exercise variables across both tests of exploration were relatively weak. When recursive partitioning was used to evaluate the predictive power of different exploration variables, novel tank diving variables were better predictors than open field variables.

Correlative analyses across different tests that measure the same behavioral trait is fundamental to understanding the underlying validity of the tests in question. In the current study, novel tank diving variables and open field variables were only weakly correlated. A plausible explanation for this is that the behavioral patterns associated with these tests are independent of each other and measure different behavioral traits (Carter et al., 2013; Perals et al., 2017). Historically, the open field test was developed for measuring activity and exploration in rodents (Hall and Ballachey, 1932), however, it has also been suggested to measure aspects of anxiety, fear, and boldness (for review, see Walsh and Cummins, 1976) or a combination of several different traits (exploration/curiosity versus fear/anxiety) (Carter et al., 2013). Similar to rodents, the open field test has been adapted for use in an aquatic setting to measure activity and exploration in different fish species including zebrafish (Champagne et al., 2010; Stewart et al., 2012), however, it has also been described as a valid test for measuring anxiety-like behavior (Godwin et al., 2012). It has even been suggested that specific variables in the open field test may correspond to different traits such that time spent in the center of the arena is the best index of exploration, amount of time frozen is the best indicator of fear, and amount of time spent around the edge of the arena is indicative of anxiety (Walsh and Cummins, 1976). The novel tank diving test was first developed by Gerlai et al. (2000) to measure swimming activity (distance and location) but has since been extensively validated as a test of anxiety-like behavior in zebrafish using anxiolytic drugs, where the amount of time spent at the bottom of the tank over the course of the trial is an indication of anxiety level (Levin et al., 2007; Cachat et al., 2011). Thus, there is much overlap in the defining purpose of the open field and novel tank diving tests, and it may be the context to which they are applied as well as the sensitivity of the variables measured that are the differential factors (Maximino et al., 2012). In the current study, the strongest correlation between test variables were those associated with freezing behavior (freezing duration in the novel tank diving test and the proportion of time frozen in the open field test). Where the results of the open field and the novel tank diving test have been directly compared, consistent behavioral patterns in freezing behavior have been found across different zebrafish strains, which was suggested to be indicative of robust anxiety-like behavior across similar contexts (Godwin et al., 2012). In addition, proportion of time frozen and movement rate had the strongest relationship in the open field test (−0.68), further highlighting the importance of freezing behavior as a sensitive indicator in the current study. Baker et al. (2018) reported that amount of time spent frozen was the most repeatable and reliable measure for assessing individual differences in the open field, and several toxicology studies have emphasized the importance of freezing behavior as a diagnostic tool in anxiolytic drug response (Grossman et al., 2010; Maximino et al., 2010) as well as erratic swimming (Maximino et al., 2010), which was not measured in the current study.

Our results show that the novel tank diving variables were more correlated with themselves than the open field variables were to each other, perhaps indicating that the novel tank diving test is a more robust behavioral test. Grossman et al. (2010) found that LSD-treated zebrafish exhibited strong responses in the novel tank diving test (decreased bottom dwelling and reduced freezing), but only evoked mild thigmotaxic behavior in the open field test. Studies using the novel tank diving test in neurobehavioral studies have reported similarly high correlations between variables (Cachat et al., 2010). Furthermore, time spent in the top of the tank had the strongest correlation with other variables suggesting that it is potentially the most valuable measurement in this test. A limitation to our study is that we did not alternate the order in which we tested fish in the exploration assays. It has been reported that following an acute stressor (predator odor exposure) transgenic mice show an increase in anxiety-like behaviors in the light dark test (Harris et al., 2022). Thus, in the current study it could be argued that behavior in the novel tank diving test was influenced by acute stress from prior testing in the open field test. However, Fontana et al. (2022) found testing order did not influence behavioral responses in the novel tank diving test and light-dark test (an alternative test of anxiety-like behavior in zebrafish). In addition, zebrafish are a shoaling species and social interaction is an important and highly developed behavior (Miller and Gerlai, 2011). However, it has been reported that acute (24 h) isolation has no effect on locomotor activity and has weak non-significant effects on anxiety-like behavior compared to chronic (6 months) of social isolation in adult zebrafish (Shams et al., 2017). The fish in the current study were isolated for 4 days in total (2 days prior to exercise observations and a further 2 days of social isolation during behavioral observations). Thus, we argue that the stress from acute (4 days) of social isolation would have had minimal effects on their exercise behavior.

In the current study we observed mild differences in the freezing behavior of males and females across the open field and novel tank diving test; males that froze for longer or spent less time moving in the novel tank diving test were more likely to display freezing behavior in the open field test compared to females. Furthermore, variables within each test were more strongly correlated overall in males than in females. Sexually dimorphic behavior has been widely reported in different animals and is commonly observed in many behavioral tests on rodent models including tests of anxiety (Johnston and File, 1991). In addition, behavioral variability between males and females can be associated with differences in strain and age (Gould et al., 2009). Despite this, pharmacological studies using animal models of human disease often do not include females because researchers fear that the estrous cycle may confound expected results (Kokras and Dalla, 2014). The role of sex in individual differences has been documented in zebrafish, but results are often conflicting (for review, see Genario et al., 2020). For example, Tran and Gerlai (2013) provided evidence to suggest that wild-type females have more consistent individual differences in total distance traveled across time and testing context, but males exhibited more robust differences in the same metric within the open field test. Mustafa et al. (2019) found no sex differences in wild-type zebrafish, but there were pronounced differences in mutant fish. Thus, the results from the current study are in partial agreement with Tran and Gerlai (2013), but more research is needed to fully understand if sex-dependency of individual differences is task specific.

In terms of physical activity, movement between the barren and swim zones was positively correlated; fish that exhibited a high number of switches into the swim zone, also exhibited a high number of switches into the barren zone. However, the more time a fish spent in the swim zone, the less time it spent in the barren zone, though this was a relatively weak association (−0.2). Therefore, even though some fish were exhibiting high amounts of movement between both zones this was not indicative of how much time the fish spent inside each zone. Novel tank diving variables had stronger positive correlations with the time in swim zone and time spent swimming than open field variables, suggesting that less-anxious fish were more motivated to be in the swim zone. High levels of exploration in the open field test have been associated with increased wheel running in different inbred strains of mice (Careau et al., 2012) and slow sprinters in wild rats (Agnani et al., 2020). In the current study, the relationship between novel tank diving variables and swimming behavior were relatively weak, thus more evidence is needed to further corroborate these findings. Moreover, there were inconsistent behavioral patterns in time spent in the different zones across different time points of the study. This could be due to a couple of different factors. Firstly, behaviors were recorded in morning and afternoon sessions, however, zebrafish activity and cortisol levels are known to fluctuate throughout the day (Cachat et al., 2010). Secondly, fish were only given 2 days to acclimate to the experimental exercise tank before recordings started. Furthermore, we only recorded behaviors across a 2-day period. Interestingly, time spent swimming was more consistent across time points on day 2 of testing. Therefore, we speculate that if we had extended the observation period a few more days we may have seen more consistent behavioral patterns.

To identify if the different exploration measures were predictive of exercise behavior, and which variables were the strongest predictors, we used a form of machine learning called recursive partitioning. Machine learning has recently come to light as an effective tool for investigating animal behavior due to its ability to deal with large, multi-dimensional and often complex data sets (for review, see Valletta et al., 2017). In the current study, recursive partitioning selected three novel tank diving variables (entries top/bottom, movement rate, and average top entry duration) and one open field variable (proportion of time spent frozen) as good predictors of exercise behavior. The higher correlation within novel tank diving variables than within open tank variables is likely to contribute to this imbalance in predictive power. Number of entries into top/bottom in the novel tank diving test was the best predictor of time spent in swim zone and time spent swimming; individuals that had more entries into top/bottom spent more time in the swim zone and more time swimming. Thus, we can conclude that individuals who were more exploratory in the novel tank diving test (as suggested by increased movement between top/bottom), spent more time in the swim zone. Contrary to our predictions, individuals that made fewer entries into the top/bottom in the novel tank diving test (i.e., exhibited less exploration), but that exhibited more freezing behavior in the open field test were predicted to spend more time in swim zone (52 s) than individuals that spent less time frozen (30 s). This suggests that individuals at the extreme ends of each behavioral trait (high amounts of freezing vs. high amounts of exploration) have a higher motivation to be in the swim zone. This motivation could be related to the natural history of zebrafish; field and lab studies on zebrafish have shown a preference for slow-moving water (McClure et al., 2006; DePasquale et al., 2019). However, it is important to point out that recursive partitioning selected an extremely low value (0.029) as the split for the proportion of time spent frozen, which is probably driven by a handful of large outliers as most individuals spent zero time frozen. Furthermore, our relatively low sample size (48 individuals) for machine learning analysis meant that the model may have needed more information to accurately predict individuals that made fewer top/bottom entries.

In conclusion, we attempted to use two different tests of exploration to predict exercise behavior in zebrafish; our results suggest that the novel tank diving test is a better predictor of exercise behavior. However, future studies should alternate the order in which fish were tested in the exploration assays to determine if prior testing experience is playing a role in the weak correlations observed in the open field variables. Moreover, our study could have benefited from the use of automatic tracking software to increase the robustness of the data and expand the behavioral repertoire to include variables that were lacking in this study (e.g., erratic swimming behavior). Finally, we believe the machine learning data analysis techniques, such as recursive partitioning, are valuable for teasing apart the complex relationships of behavioral data.

## Data availability statement

The data that support the findings of this study are openly available in Dryad at https://doi.org/10.5061/dryad.c2fqz61c8.

## Ethics statement

The animal study was reviewed and approved by the IACUC at the Pennsylvania State University (protocol 201900937).

## Author contributions

CD and KF conceived the original idea. CD supervised the project. KF and ZJ conducted the behavioral experiments. KF, ZJ, and KJ analyzed the videos. FB conducted the statistical analyses and wrote the statistical methods and results. CD wrote the introduction, methods, and discussion with support from KF, ZJ, KJ, and FB. All authors contributed to the article and approved the submitted version.
