# Supplementary material for: The effects of exploratory behavior on physical activity in a common animal model of human disease, zebrafish (Danio rerio)
Source: Front Behav Neurosci. 2022 Nov 8;16:1020837. doi: 10.3389/fnbeh.2022.1020837 (PMC9679429; doi:10.3389/fnbeh.2022.1020837)
Supplement: Supplementary file 1 [file Data_Sheet_1.docx]

Supplemental Material

Supplemental Table 1. A priori predictions of correlations between variables within the novel tank diving test, within the open field test, and between the novel tank and open field tests. Italicized rows are those in which the direction of the prediction was supported, and rows are bolded if the correlation coefficient was statistically significant at an α-value of 0.05 (even if the direction was not as predicted). For this visualization, we used the results from analyses of the data from all individuals and not the sex-specific or time-varying results.

| A priori predictions of correlations within novel tank diving variables | | |
| --- | --- | --- |
| Variable 1 | Variable 2 | Positive (+) or Negative (-) Relationship |
| ***Latency to Top (s)*** | ***Time in Top (s)*** | ***-*** |
| ***Latency to Top (s)*** | ***Time in Top/Bottom*** | ***-*** |
| ***Time in Top (s)*** | ***Time in Top/Bottom*** | ***+*** |
| ***Entries to Top*** | ***Entries to Top/Bottom*** | ***+*** |
| **Entries to Top** | **Av. Top Entry Duration (s)** | **-** |
| ***Freeze Duration (s)*** | ***Movement Rate*** | ***-*** |
| ***Entries to Top*** | ***Movement Rate*** | ***+*** |
| *Entries to Top/Bottom* | *Movement Rate* | *+* |
| ***Freeze Duration (s)*** | ***Entries to Top*** | ***-*** |
| ***Freeze Duration (s)*** | ***Entries to Top/Bottom*** | ***-*** |
| A priori predictions of correlations within open field variables | | |
| Variable 1 | Variable 2 | Positive (+) or Negative (-) Relationship |
| ***Latency to edge (s)*** | ***Prop. of Time in Center*** | ***+*** |
| ***Prop. of Time in Center*** | ***Av. Center Duration (s)*** | ***+*** |
| ***Movement Rate*** | ***Prop. of Time Spent Frozen*** | ***-*** |
| A priori predictions of correlations between novel tank (NT) and open field (OF) variables | | |
| Variable 1 (NT) | Variable 2 (OF) | Positive (+) or Negative (-) Relationship |
| *Time in Top (s)* | *Av. Center Duration (s)* | *+* |
| *Time in Top/Bottom* | *Av. Center Duration (s)* | *+* |
| Time in Top (s) | Prop. of Time in Center | + |
| *Entries to Top* | *Movement Rate* | *+* |
| *Entries to Top/Bottom* | *Movement Rate* | *+* |
| *Av. Top Entry Duration (s)* | *Av. Center Duration (s)* | *+* |
| ***Freeze Duration*** | ***Movement Rate*** | ***-*** |
| ***Freeze Duration*** | ***Prop. of Time Frozen*** | ***+*** |
| ***Movement Rate*** | ***Prop. of Time Frozen*** | ***-*** |
| ***Movement Rate*** | ***Movement Rate*** | ***+*** |


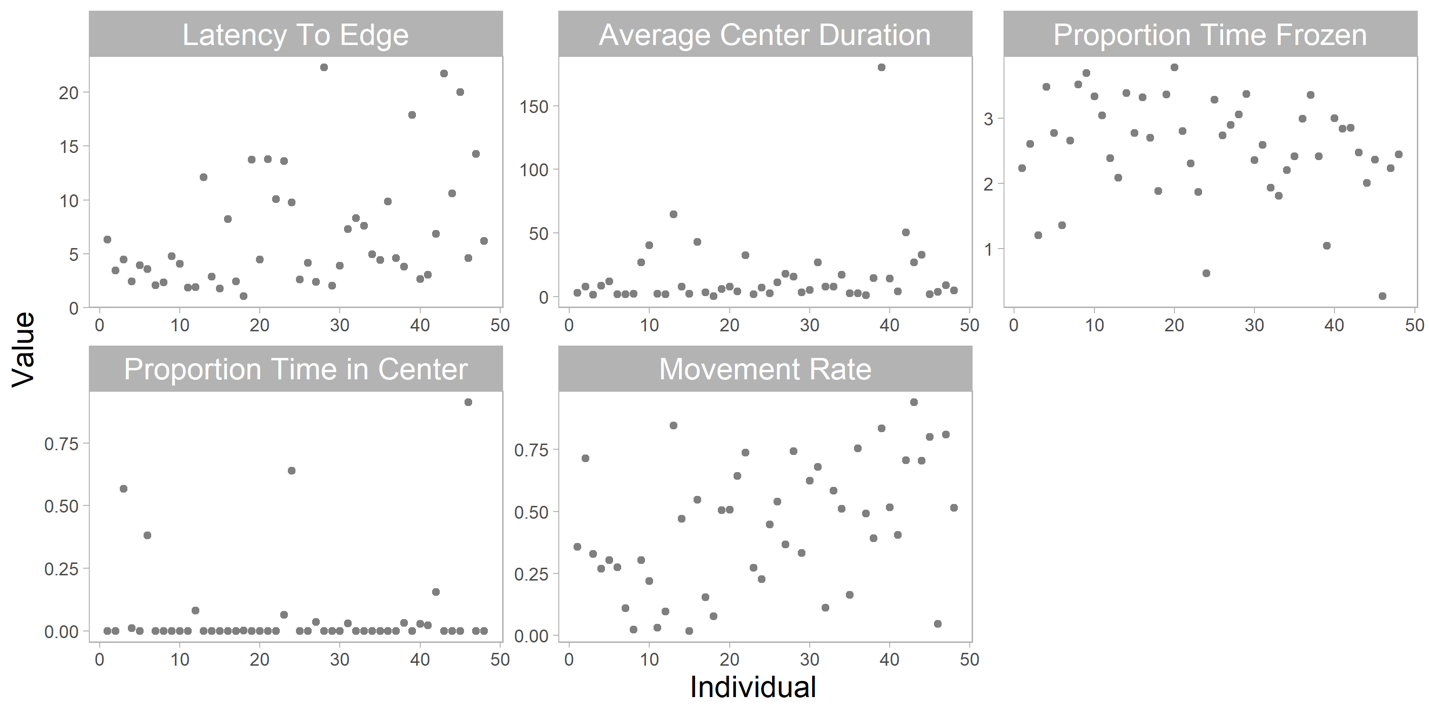


Supplemental Figure 1: The values for each individual fish across each variable in the open field test.


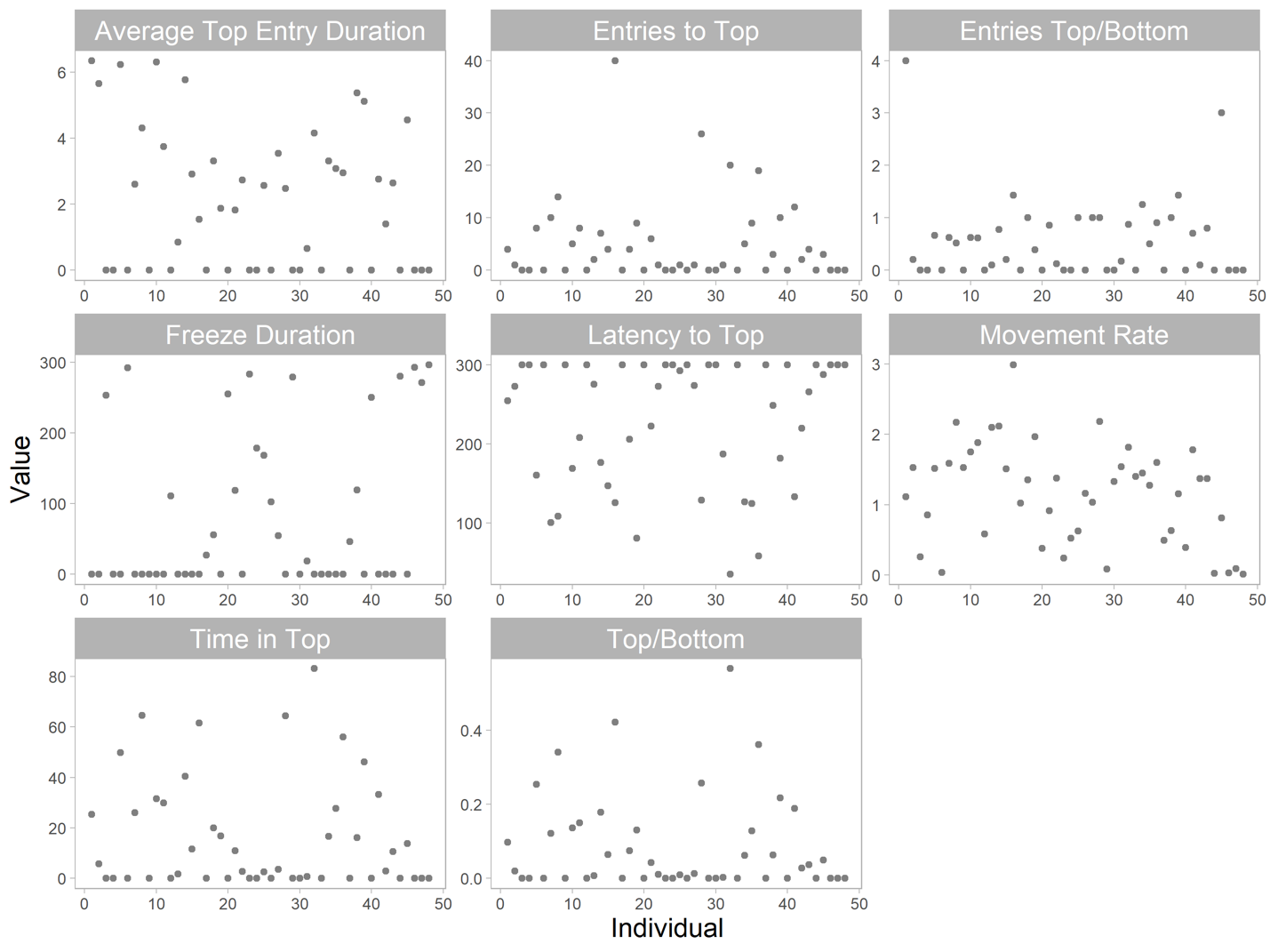


Supplemental Figure 2: The values for each individual fish across each variable in the novel tank diving test.


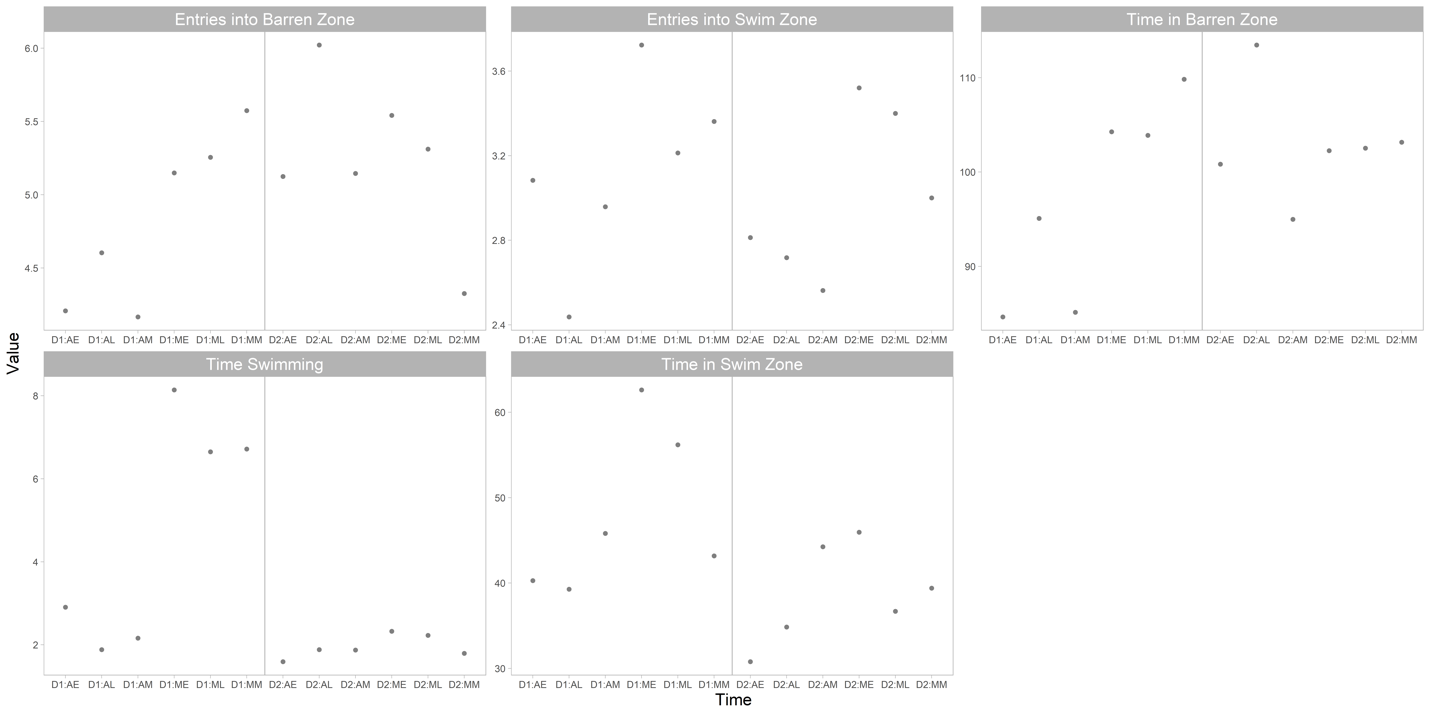


Supplemental Figure 3: The average value across individuals for each exercise variable at each time-point.


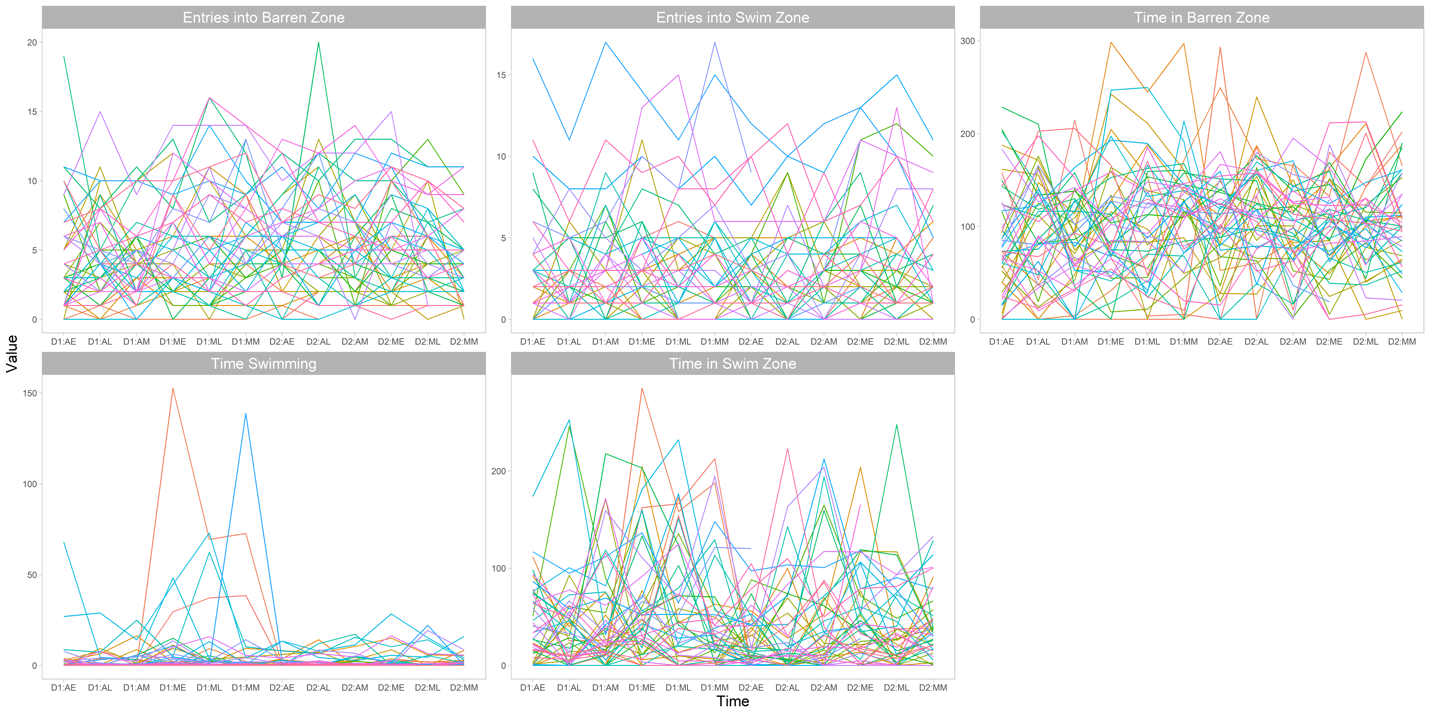


Supplemental Figure 4: A comparison of the average exercise variables (across time) for time in swim zone and time spent swimming for each individual.
